# Transient Receptor Potential Channels as Key Regulators of Neuroinflammation in Neurological Disorders: Mechanistic Insights, Therapeutic Potentials, and Future Directions

**DOI:** 10.1002/cns.70700

**Published:** 2025-12-15

**Authors:** Daji Guo, Mengjiao Cai, Yanmin Xian, Yinyin Chen, Yujun Feng, Chun Hu, Lizhang Zeng, Lei Shi, Shiqing Zhang

**Affiliations:** ^1^ Key Laboratory of Brain, Cognition and Education Science, Ministry of Education South China Normal University Guangzhou China; ^2^ Institute for Brain Research and Rehabilitation South China Normal University Guangzhou China; ^3^ State Key Laboratory of Bioactive Molecules and Druggability Assessment, Guangdong Basic Research Center of Excellence for Natural Bioactive Molecules and Discovery of Innovative Drugs Jinan University Guangzhou China; ^4^ JNU‐HKUST Joint Laboratory for Neuroscience and Innovative Drug Research, College of Pharmacy Jinan University Guangzhou China; ^5^ Guangdong Province Key Laboratory of Pharmacodynamic Constituents of TCM & New Drugs Research, Guangdong Hong Kong‐Macau Joint Laboratory for Pharmacodynamic Constituents of TCM and New Drugs Research Jinan University Guangzhou China

**Keywords:** neuroinflammation, neurological disorders, neuroprotection, therapeutic strategy, transient receptor potential ion channels

## Abstract

**Background:**

Transient receptor potential (TRP) ion channels, a ubiquitous family of nonselective cation channels, are extensively expressed across the nervous system, immune system, and peripheral tissues. These channels serve as critical sensors for detecting temperature, mechanical forces, and chemical stimuli, thereby regulating numerous physiological and pathological processes. Over the past decade, their pivotal role in neuroimmune crosstalk and inflammatory signaling has emerged as a key focus within neuroscience research.

**Methods:**

A comprehensive literature review was conducted in PubMed using key terms “TRP channel,” “neuroinflammation,” and each of the following neurological disorders: neuropathic pain, migraine, stroke, multiple sclerosis (MS), Alzheimer's disease (AD), Parkinson's disease (PD), autism spectrum disorder (ASD), epilepsy, and psychiatric disorders.

**Results:**

This review synthesizes the current evidence to elucidate the dual‐edged contributions of TRP channels as mediators of inflammation in neuropathic pain, migraine, stroke, MS, AD, PD, ASD, epilepsy, and psychiatric disorders. Furthermore, we also evaluate emerging therapeutic strategies targeting TRP channels, encompassing both nonpharmacological approaches and pharmacological interventions.

**Conclusions:**

By integrating mechanistic insights with translational perspectives, this review highlights TRP channels as promising targets for precision medicine and underscores their potential in the development of novel, mechanism‐based therapies for complex neurological disorders, thereby advancing a new era of targeted neuroimmunomodulation.

## Graphical abstract

This study elucidates the central role of transient receptor potential (TRP) channels in driving neuroinflammation across neurological disorders. Acting as critical molecular sensors on neurons and glial cells, TRP channels' activation initiates downstream pathways that promote the release of proinflammatory cytokines, a pathological mechanism in neurological disorders. Modulation of TRP channel activity can attenuate neuroinflammation and alleviate disease progression, highlighting the therapeutic potential of TRP channels.

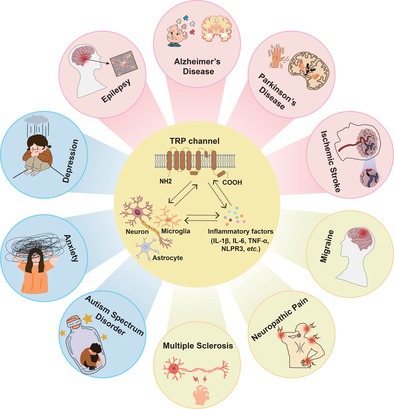

**Abbreviations**
2‐APB: 2‐aminoethoxydiphenyl borate
6‐OHDA: 6‐hydroxydopamine
α‐syn: α‐synuclein
ACA: N‐(p‐amycinnamoyl) anthranilic acid
ACEA: arachidonyl‐2'‐chloroethylamide
AD: Alzheimer's disease
ADP: adenosine diphosphate
AEA: anandamide
AMPK: adenosine 5′‐monophosphate (AMP)‐activated protein kinase
APOE ε4: apolipoprotein E ε4
ASC: apoptosis‐related spotted protein
ASD: autism spectrum disorder
Aβ: amyloid‐β
BAFF: B‐cell activating factor
BBB: blood–brain barrier
BCCAO/R: bilateral common carotid artery occlusion followed by reperfusion
BCP: beta‐caryophyllene
BDNF: brain‐derived neurotrophic factor
BoNTs: botulinum toxin
CaMKII: calcium/calmodulin‐dependent protein kinase II
Caspase‐1: cysteinyl aspartate‐specific protease‐1
CB: cannabinoid receptor
CBA: 4‐Chloro‐2‐(2‐(2‐chlorophenoxy) acetamido) benzoic acid
CBD: cannabidiol
CCL: CC‐Chemokines ligand
CCI: chronic constriction injury
CFA: complete Freunde's adjuvant
CGRP: calcitonin gene‐related peptide
CREB: cyclic‐AMP response binding protein
CSPGs: chondroitin sulfate proteoglycans
CNS: central nervous system
COX‐2: cyclooxygenase‐2
CRAP: coumarins from Radix angelicae *pubescentis*
CS‐AT NPs: Cu₂₋ₓSe‐anti‐TRPV1 nanoparticles
CSF: cerebrospinal fluid
CXCL: C‐X‐C motif chemokine ligand
DA: dopamine
DRG: dorsal root ganglion
EA: electroacupuncture
EAE: experimental autoimmune encephalomyelitis
ER: endoplasmic reticulum
ERK: extracellular regulated protein kinase
FAF1: Fas‐associated factor 1
FDA: Food and Drug Administration
GFRα3: neurotrophic factor family receptor alpha‐3
GSK3: glycogen synthase kinase‐3
HMGB1: high mobility group box 1
IL‐: interleukin‐
iNOS: inducible nitric oxide synthase
iRTX: 5′‐iodoresiniferatoxin
JNK: c‐Jun N‐terminal kinase
KA: kainic acid
LSC: lignans of *Schisandra chinensis* (Turcz.) Baill
MAPK: mitogen‐activated protein kinase
MCAO/R: middle cerebral artery occlusion reperfusion
MHC II: major histocompatibility complex II
MPTP: 1‐methyl‐4‐phenylpyridinium
MS: multiple sclerosis
NAc: nucleus accumbens
NBA: 4‐Chloro‐2‐(2‐(naphthalene‐1‐yloxy) acetamido) benzoic acid
NECAB2: N‐Terminal EF‐Hand Ca^2+^ Binding Protein 2
NF‐κB: nuclear factor kappa‐B
NFTs: neurofibrillary tangles
NG: nodose ganglion
NGF: nerve growth factor
NLRP3: nucleotide‐binding oligomerization domain‐like receptor pyrin domain containing 3
NOS2: nitric oxide synthase 2
OGD/R: oxygen and glucose deprivation/reoxygenation
PBMC: 1‐phenylethyl‐4‐(benzyloxy)‐3‐methoxybenzyl (2‐aminoethyl) carbamate
PD: Parkinson's disease
PFC: the prefrontal cortex
PI(3,5)P_2_
: phosphatidylinositol 3,5‐bisphosphate
PKM2: pyruvate kinase M2
PLC: phospholipase C
PP2A: protein phosphatase 2A
PSNL: partial sciatic nerve ligation
ROS: reactive oxygen species
rTMS: repetitive transcranial magnetic stimulation
RTX: resiniferatoxin
SN: substantia nigra
SNI: spared nerve injury
SNP: single‐nucleotide polymorphism
SREBP: sterol regulatory element‐binding protein
STAT3: signal transducer activator of transcription 3 signaling
TCA: trans‐cinnamaldehyde
TENS: transcutaneous electrical nerve stimulation
TG: trigeminal ganglion
TGF‐β: transforming growth factor β
TLR: Toll‐like receptor
TNC: trigeminal nucleus caudalis
TNF‐α: tumor necrosis factor α
TRP: transient receptor potential
TRPA: transient receptor potential ankyrin subfamily
TRPC: transient receptor potential canonical subfamily
TRPM: transient receptor potential melastatin subfamily
TRPML: transient receptor potential mucin subfamily
TRPP: transient receptor potential polycystic subfamily
TRPV: transient receptor potential vanilloid subfamily
VSLD: voltage‐sensor‐like domain
XSZT: Xiongshao Zhitong granules
YAP: Yes‐associated protein

## 1 Introduction

Transient receptor potential (TRP) ion channels are a superfamily of nonselective cation channels that play critical roles in sensory transduction, cellular signaling, and homeostasis. TRP channels are widely expressed in a variety of tissues, including the nervous system, immune system, and peripheral tissues, and play an important role in various physiological and pathological processes [1]. Increasing lines of evidence highlight TRP channels as key regulators of neuroinflammation, a complex and important pathological process in multiple neurological diseases that encompasses a broad range of immune responses in the central nervous system (CNS). This process involves the activation of glia, release of cytokines and chemokines, disruption of vascular permeability, and infiltration of leukocytes, collectively driving neuronal dysfunction and neurodegeneration. In this review, we shed light on the role of TRP channel family members in regulating neuroinflammation. Key TRP channel subtypes, including TRPA1, TRPC1, TRPC3, TRPC6, TRPM2, TRPM4, TRPM7, TRPM8, TRPV1, TRPV2, and TRPV4, are discussed with an emphasis on their roles in mediating the pathophysiology of several neurological disorders. These ion channels emerge as critical mediators of inflammatory and neurotoxic processes in diseases such as neuropathic pain, migraine, stroke, multiple sclerosis (MS), Alzheimer's disease (AD), Parkinson's disease (PD), autism spectrum disorder (ASD), epilepsy, and psychiatric disorders. We further review and discuss the therapeutic strategies to modulate TRP channels, including both pharmacological approaches (agonists/antagonists) and nonpharmacological interventions (neuromodulation). This review positions TRP channels as both biomarkers of neuroinflammatory pathology and actionable targets for neurological diseases.

## 2 A Brief Glance of TRP Channels

TRP ion channels play critical roles in sensing environmental stimuli and modulating cellular responses. This family was initially identified in fruit flies due to its role in photoconduction and was later found to be involved in a variety of normal and disease‐related processes [1]. They are conserved across mammalian species and exhibit remarkable structural diversity, enabling them to respond to a wide range of stimuli, including temperature, mechanical force, pH, and chemical ligands. TRP channels are tetrameric proteins, with each subunit typically containing six transmembrane helices (S1–S6) flanked by intracellular N‐ and C‐termini [1, 2]. S1–S4 form a voltage‐sensor‐like domain (VSLD), though most TRP channels are not voltage‐gated. S5–S6 and the intervening pore loop form the ion‐conducting pore. The N‐terminus of TRP channels varies in length and composition, often containing regulatory domains such as the ankyrin repeats and coiled‐coil regions, while the C‐terminus possesses motifs critical for channel assembly, trafficking, and interaction with signaling molecules (e.g., TRP box, calmodulin‐binding sites) [1, 2]. The TRP family consists of 28 members in rodents and 27 members in humans, with only TRPC2 absent [3]. They are divided into six subfamilies based on their protein sequence homology and structural features, namely, TRPA (ankyrin, TRPA1), TRPC (canonical protein, TRPC1–7), TRPM (melastatin, TRPM1–8), TRPML (mucolipin, TRPML1–3), TRPP (polycystic protein, TRPP2, 3, and 5), and TRPV (vanilla protein, TRPV1–6) [2, 3].

**FIGURE 1:**
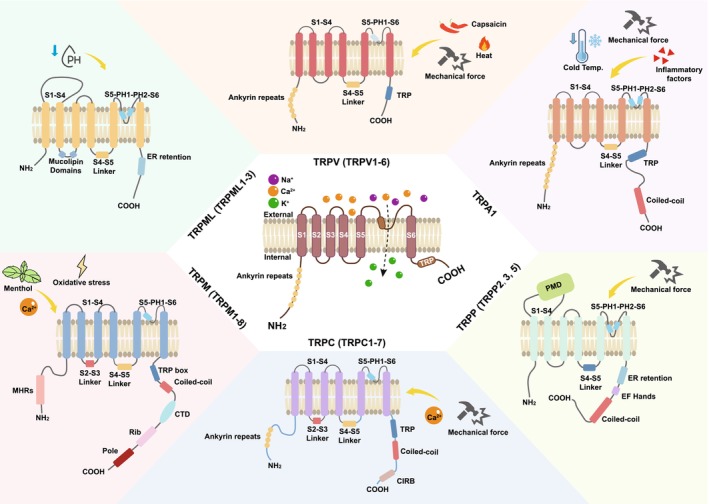
Structures of different TRP channel subfamilies. Center: Representative structure of the TRP channels. Outer: Representative structures of different TRP channel subfamilies as indicated.

Numerous reviews have summarized that each TRP subfamily exhibits unique structural and functional features (Figure 1) [1, 2, 3]. TRPA1, the sole mammalian TRPA member, has an exceptionally long N‐terminus with 14–17 ankyrin repeats critical for mechanical/chemical sensing and a non‐selective cation pore activated by reactive electrophiles, noxious cold, and inflammatory mediators [4]. TRPC channels (TRPC1–7), primarily activated via Gq‐coupled receptors and phospholipase C (PLC), feature 3–4 ankyrin repeats in their N‐terminus for protein–protein interactions, a C‐terminus with a conserved TRP box (EWKFAR), and calmodulin/IP3 receptor‐binding domains, and a pore favoring Ca^2+^ and Na^+^ permeability [5]. TRPM channels (TRPM1–8) have a long (~700 residues) N‐terminus with a unique TRPM homology region, C‐terminal coiled‐coil domains and enzymatic motifs, and pores with low Ca^2+^ selectivity (except TRPM4/5, which are monovalent‐selective). TRPM2, TRPM4/5, and TRPM8 are activated by oxidative stress, intracellular Ca^2+^, and cold, respectively [6]. TRPML channels (TRPML1–3) reside in lysosomes/endosomes, featuring short extracellular N‐termini, large intracellular loops, and highly Ca^2+^‐selective pores oriented “luminally.” They are regulated by phosphatidylinositol 3,5‐bisphosphate (PI(3,5)P_2_) and pH changes, playing roles in lysosomal ion homeostasis and trafficking [7]. TRPP channels, including TRPP2, 3, and 5, have 6‐transmembrane motifs in the extracellular N‐terminus. TRPP2 forms heteromeric complexes for mechanosensation in renal tubules and cilia, with Ca^2+^ and K^+^ permeability [3]. TRPV members (TRPV1–6) display structural variability, with 3–6 ankyrin repeats in the N‐terminus (TRPV1–4) or shorter N‐termini (TRPV5/6), a wider pore region (highly Ca^2+^‐selective in TRPV5/6), and C‐terminal TRP boxes in TRPV1–4. These channels respond to diverse stimuli such as heat (TRPV1 and TRPV2), osmolarity (TRPV4), and endogenous lipids [1, 8, 9, 10]. Each TRP subfamily thus integrates specific structural motifs and activation mechanisms to regulate cellular processes such as sensory perception, ion homeostasis, and mechanotransduction. Ion selectivity is primarily determined by the residues in the pore loop region. For example, TRPV5 and TRPV6 feature an aspartate residue that confers their high selectivity for Ca^2+^. TRPV1 and TRPM8 utilize specialized S1–S4 domains to function as temperature sensors, enabling thermosensation. The ankyrin repeats in TRPA1 and the extracellular domains in TRPP1 are crucial for sensing mechanical force. Additionally, ligand binding is a key aspect of TRP channel activation. TRPV1 has a capsaicin‐binding site located between the S3 and S4 transmembrane segments, while TRPM2 binds ADP‐ribose through its C‐terminal enzymatic domain.

Within the nervous system, TRP channel members are distributed across multiple cell types, including neurons, glial cells, and endothelial cells. Members of the TRPA, TRPC, TRPM, and TRPV channels are expressed in neurons of different brain regions, such as the cortex, hippocampus, thalamus, spinal cord. Notably, in the dorsal root ganglion (DRG) neurons, TRPA1, TRPC1/3/6, TRPM2/7/8, TRPV1/4 act as sensors for pain, temperature, and mechanical pressure [3, 11]. In microglia, TRPC1/3/6, TRPM2/4/7/8, and TRPV1/2/4 channels contribute to processes such as proliferation, migration, and cytokine production [12, 13, 14, 15, 16]. TRPA1, TRPC1–6, TRPM2/7, TRPV1/2/4 expressed in astrocytes participate in maintaining blood–brain barrier (BBB) function, GABA release, and neuroinflammation [17, 18, 19, 20]. Beyond these cell types, only a small subset of TRP channels (TRPA1, TRPC3, and TRPV3) are expressed in oligodendrocytes [21, 22], while TRPC1, TRPM3, and TRPV2 in oligodendrocyte precursor cells (OPC) modulate their proliferation or myelination capacity [23, 24, 25]. Additionally, TRPA1, TRPC1/3/6, TRPM2, TRPV1/4 channels in brain endothelial cells are found to be involved in inflammatory processes [26].

## 3 TRP Channels in Regulating Neuroinflammation

**FIGURE 2:**
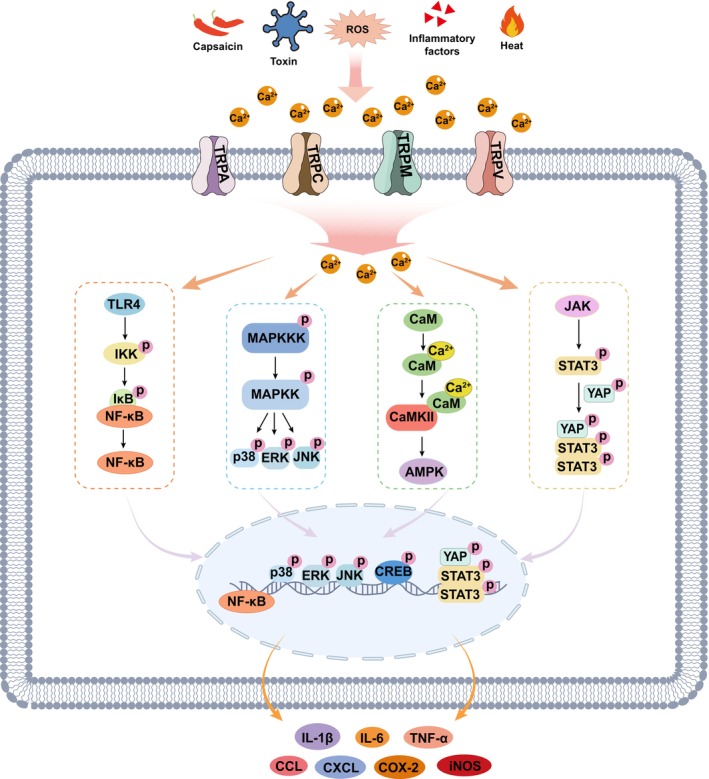
A schematic figure illustrates the intracellular signaling pathways mediating cytokine release downstream of TRP activation. Upon sensing extracellular stimuli (e.g., heat, ROS, toxins, or capsaicin), TRP channels open and trigger a rapid rise in intracellular Ca^2+^. This Ca^2+^ signal sequentially activates the TLR4‐NF‐κB, MAPK‐MAPKK, CaMKII‐AMPK, and JAK‐STAT3 cascades, culminating in the transcriptional release of proinflammatory cytokines (e.g., IL‐1β, IL‐6, TNF‐α, COX‐2, CXCLs, CCLs, and iNOS). AMPK, adenosine 5′‐monophosphate (AMP)‐activated protein kinase; CaMKII, calcium/calmodulin‐dependent protein kinase II; CCL, CC‐Chemokines ligand; COX‐2, cyclooxygenase‐2; CREB, cyclic‐AMP response binding protein; CXCL, C‐X‐C motif chemokine ligand; ERK, extracellular regulated protein kinase; IL‐, interleukin‐; iNOS, inducible nitric oxide synthase; IKK, inhibitor of kappa B kinase; JAK, Janus kinase; JNK, c‐Jun N‐terminal kinase; NF‐κB, nuclear factor kappa‐B; MAPK, mitogen‐activated protein kinase; ROS, reactive oxygen species; STAT3, signal transducer activator of transcription 3; YAP, Yes‐associated protein.

TRP channel activation plays an important regulatory role in neuroinflammation primarily by mediating Ca^2+^‐signaling, which influences glial activation, cytokine release such as interleukin‐ (IL‐) 1β, tumor necrosis factor α (TNF‐α), and IL‐6, and BBB integrity. Members of the TRPA, TRPC, TRPM, and TRPV subfamilies are involved in neuroinflammatory responses by regulating the release of proinflammatory mediators and influencing neuronal excitability. Across diverse pathophysiological contexts, activation of TRP channels predominantly exacerbates the inflammatory response through multiple pathways, including adenosine 5′‐monophosphate (AMP)‐activated protein kinase (AMPK), cyclic‐AMP response binding protein (CREB), extracellular regulated protein kinase (ERK), c‐Jun N‐terminal kinase (JNK), nuclear factor kappa‐B (NF‐κB), p38 mitogen‐activated protein kinase (MAPK), and signal transducer activator of transcription 3 signaling (STAT3) (Figure 2). For instance, TRPM2 or TRPM8 activation promotes proinflammatory cytokine (IL‐1β, IL‐6, and TNF‐α) release with increasing Ca^2+^ concentration and the activation of p38 MAPK, JNK, and NF‐κB [27, 28, 29]. TRPV1 activation induces Ca^2+^ influx and then promotes the activation of p38 MAPK, followed by the release of IL‐1β, IL‐6, IL‐8, and TNF‐α [30, 31]. In an inflammatory pain rat model, activation of TRPV1 in sensory neurons leads to the release of neuropeptides such as calcitonin gene‐related peptide (CGRP) through calcium/calmodulin‐dependent protein kinase II (CaMKII) and CREB signaling in DRG, thereby amplifying the inflammatory response [32]. Increased TRPV1 expression induces ERK phosphorylation and cytokine expression during pain pathophysiology [33]. Moreover, elevated TRPV1 or TRPV4 promotes the inflammatory response through the Toll‐like receptor 4 (TLR4)‐NF‐κB signaling pathway [34, 35], and upregulated TRPV4 activity elevates intracellular Ca^2+^, promoting the phosphorylation of Yes‐associated protein (YAP) and STAT3 and subsequent proinflammatory cytokine production [36]. In PD pathology, downregulated TRPV1 inhibits AMPK signaling and induces the expression of IL‐1β, inducible nitric oxide synthase (iNOS), and cyclooxygenase‐2 (COX‐2) [37]. TRPA1 channel, activated by reactive oxygen species (ROS) and other inflammatory byproducts, enhances inflammation response‐mediated neuronal damage via the MAPK/NF‐κB pathway [4]. Inhibited TRPA1 activity downregulates Ca^2+^ influx and NF‐κB activity, attenuating cytokine release in AD mice [38]. However, TRPC6 activation reduces IL‐1β and IL‐6 release and NF‐κB activity in an in vitro ischemic stroke model, partly via inhibiting Ca^2+^ signaling [39].

Neuroimmunological studies reveal the central role of glial cells in immune regulation within the CNS [40, 41]. Microglia, the CNS‐resident immune cells, detect pathogens or damage via receptors such as TLRs and nucleotide‐binding oligomerization domain‐like receptor pyrin domain containing 3 (NLRP3), releasing proinflammatory cytokines (e.g., TNF‐α, IL‐1β) and phagocytosing debris to drive neuroinflammation. Astrocytes recruit peripheral immune cells by secreting chemokines (e.g., CC‐Chemokines ligand [CCL] 2 and C‐X‐C motif chemokine ligand [CXCL] 10) and form protective glial scars. Their interplay is dynamic: microglial IL‐1α/TNF‐α induces neurotoxic A1 astrocytes, while anti‐inflammatory microglia (e.g., TREM2‐dependent) promote neuroprotective A2 astrocytes. Dysregulation of this balance exacerbates multiple neurological diseases. TRP channels in astrocytes or microglia also participate in these processes. The activation of the TRPV1 channel in glial cells can increase Ca^2+^ influx, prompting proinflammatory cytokines' release such as IL‐1β and TNF‐α [17]. Ca^2+^‐dependent TRPC6 activation leads to upregulated p38 MAPK signaling and increased secretion of the chemokine CXCL1 in astrocytes [42]. Given their involvement in neuroinflammation regulation, this family of ion channels has attracted considerable attention as a potential therapeutic target for the treatment of neurological diseases.

## 4 TRP Channels in Neurological Disorders

**FIGURE 3:**
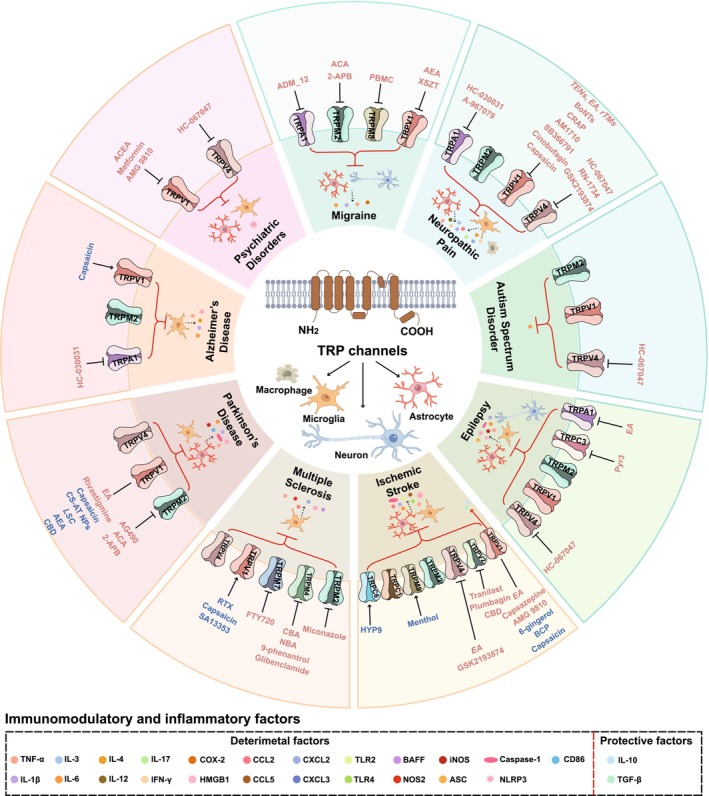
Schematic of modulatory effects toward neuroimmune responses mediated by different TRP channels in neurological disorders (neuropathic pain, migraine, stroke, multiple sclerosis, Alzheimer's disease, Parkinson's disease, autism spectrum disorder, epilepsy, and psychiatric disorders) and the targeted therapeutic strategies. Strategies that show excitatory effects to TRP channels are in blue, and those show inhibitory effects are in reddish color. Pharmacological and non‐pharmacological strategies are presented in regular or italic bold font, respectively. 2‐APB, 2‐aminoethoxydiphenyl borate; ACA, N‐(p‐amycinnamoyl) anthranilic acid; ACEA, arachidonyl‐2′‐chloroethylamide; AEA, anandamide; ASC, apoptosis‐related spotted protein; BAFF, B‐cell activating factor; BoNTs, botulinum toxin; CBA, 4‐Chloro‐2‐(2‐(2‐chlorophenoxy) acetamido) benzoic acid; Caspase‐1, cysteinyl aspartate‐specific protease‐1; CBD, cannabidiol; CCL, CC‐Chemokines ligand; COX‐2, cyclooxygenase‐2; CRAP, coumarins from Radix angelicae *pubescentis*; CS‐AT NPs, Cu₂₋ₓSe‐anti‐TRPV1 nanoparticles; CXCL2, C‐X‐C motif chemokine ligand2; EA, electroacupuncture; IL‐, interleukin‐; iNOS, inducible nitric oxide synthase; LSC, Lignans of Schisandra chinensis (Turcz.) Baill; NBA, 4‐Chloro‐2‐(2‐(naphthalene‐1‐yloxy) acetamido) benzoic acid; NLRP3, nucleotide‐binding oligomerization domain‐like receptor pyrin domain containing 3; NOS2, nitric oxide synthase 2; PBMC, 1‐phenylethyl‐4‐(benzyloxy)‐3‐methoxybenzyl(2‐aminoethyl) carbamate; rTMS, repetitive transcranial magnetic stimulation; RTX, resiniferatoxin; TENS, transcutaneous electrical nerve stimulation; TLR, Toll‐like receptors; TNF‐α, tumor necrosis factor α; XSZT, Xiongshao Zhitong granules.

Neuroinflammation has been identified as a key factor in the progression of several neurodegenerative and mental disorders, exerting detrimental effects on neurons. Emerging evidence suggests that specific members of TRP channels, including TRPA1, TRPC1, TRPC3, TRPC6, TRPM2, TRPM4, TRPM7, TRPM8, TRPV1, TRPV2, and TRPV4, are implicated in neuroinflammatory processes and play crucial roles in the pathophysiology of neuropathic pain, migraine, stroke, MS, AD, PD, ASD, epilepsy, anxiety, and depression (Figure 3 and Tables 1, 2, 3, 4, 5). In this part, we discuss the different roles of TRP channels in each disease.

**TABLE 1:** Roles of TRP channels in neurological diseases.

TRP channels		Neuropathic pain	Migraine	Ischemic stroke	MS	AD	PD	ASD	Epilepsy	Psychiatric disorders
TRPA	TRPA1	Detrimental	Detrimental			Detrimental	Detrimental	Detrimental	Detrimental	
TRPC	TRPC1			Dual role						
	TRPC3								Detrimental	
	TRPC6			Dual role						
TRPM	TRPM2	Detrimental	Detrimental	Detrimental	Detrimental	Detrimental	Detrimental	Detrimental	Detrimental	
	TRPM4				Detrimental					
	TRPM7				Detrimental					
	TRPM8		Detrimental	Protective						
TRPV	TRPV1	Detrimental	Detrimental	Dual role	Dual role	Dual role	Dual role	Protective	Detrimental	Detrimental
	TRPV2			Detrimental						
	TRPV4	Detrimental		Detrimental	Detrimental		Detrimental	Detrimental	Detrimental	Detrimental

Abbreviations: AD, Alzheimer's disease; ASD, autism spectrum disorder; MS, multiple sclerosis; PD, Parkinson's disease.

**TABLE 2:** TRPA channels in neurological diseases.

TRPA channels	Disease	Expression or activity (change in disease)	Modulatory effects in neuroinflammation	TRP‐targeted approaches that show therapeutic effects	References
TRPA1	Neuropathic pain	Upregulated	Modulates CCL2 expression in monocytes/macrophages.	*Trpa1* knockout TRPA1 inhibitors (HC‐030031, A‐967079)	[43, 44]
	Migraine	Activated by oxidative stress	Epigenetic modifications regulate TRPA1‐mediated neurogenic inflammation.	TRPA antagonist (ADM_12)	[45, 46, 47]
	AD	Activated in astrocytes by Aβ	Enhances astrocytic inflammatory responses via Ca^2+^‐signaling.	*Trpa1* knockout TRPA1 inhibitor (HC‐030031)	[38]
	Epilepsy	Upregulated in hippocampus		EA (reduces TRPA1 expression)	[48]

Abbreviations: Aβ, amyloid‐β; AD, Alzheimer's disease; CCL2, chemokine (C‐C motif) ligand 2; EA, electroacupuncture; TCA, trans‐cinnamaldehyde.

**TABLE 3:** TRPC channels in neurological diseases.

TRPC channels	Disease	Expression or activity (change in disease)	Modulatory effects in neuroinflammation	TRP‐targeted approaches that show therapeutic effects	References
TRPC1	Ischemic stroke	Upregulated or SUMOylated in microglia Decreased in brain tissues	Increases secretion of CCL5 and CCL2. Promotes NLRP3 inflammasome activation in microglia.	Microglia conditional *Trpc1* knockdown	[16, 49, 50]
TRPC3	Epilepsy	Elevated in rat hippocampal neurons	Elevates TNF‐α and IL‐1β in hippocampal neurons.	TRPC3 inhibitor (Pyr3)	[51]
TRPC6	Ischemic stroke	Decreased in the cortex and in cultured astrocytes Increased in the cortical neurons	Inhibits proinflammatory factors (IL‐6, IL‐1β), and modulates NF‐κB pathway.	TRPC6 agonist (HYP9) Overexpression of *Trpc6*	[39, 52, 53, 54]

Abbreviations: CCL, CC‐Chemokines ligand; IL‐, interleukin‐; NF‐κB, nuclear factor kappa‐B; NLRP3, nucleotide‐binding oligomerization domain‐like receptor pyrin domain containing 3; TNF‐α, tumor necrosis factor α.

**TABLE 4:** TRPM channels in neurological diseases.

TRPM channels	Disease	Expression or activity (change in disease)	Modulatory effects in neuroinflammation	TRP‐targeted approaches that show therapeutic effects	References
TRPM2	Neuropathic pain	Upregulated in immune cells or microglia	Promotes microglia activation, neutrophil infiltration and CXCL2 production. Regulates neuroinflammation via TLR4.	*Trpm2* knockout	[11, 55, 56, 57]
	Migraine	Increased in TG neurons	Modulates ROS, apoptosis, Ca^2+^ levels, and cytokines (TNF‐α, IL‐1β, IL‐6) in TG neurons.	*Trpm2* knockout TRPM2 inhibitors (ACA, 2‐APB)	[58, 59]
	Ischemic stroke	Activated	Induces apoptosis, Ca^2+^ overload, ROS, and NLRP3 inflammasome activity.	*Trpm2* knockout	[60, 61]
	MS	Activated	Activates microglia, drives NLRP3 inflammasome, and promotes chemokine release.	*Trpm2* knockout TRPM2 inhibitor (miconazole)	[62, 63]
	AD	Activated in aging neurons and glia	ROS‐induced NLRP3 inflammasome production.	*Trpm2* knockout	[64, 65, 66]
	PD	Activated	Promotes proinflammatory cytokines (IL‐1β, TNF‐α, IL‐6, iNOS, and CD86).	*Trpm2* knockout TRPM2 inhibitors (ACA, 2‐APB, AG490)	[67, 68]
	ASD	Downregulated in NG of *Shank3* knockout mice	Participates in the neuroinflammatory pathology in *SHANK3*‐related ASD.		[69]
	Epilepsy	Activated in hippocampus	Exacerbates seizures via glial activation and neuroinflammation.	*Trpm2* knockout	[70, 71]
TRPM4	MS	Upregulated	Drives proinflammatory factors release (TNF‐α, CCL2, BAFF, and NOS2).	TRPM4 inhibitors (9‐phenantrol, glibenclamide, CBA, NBA) *Trpm4* knockout	[72, 73, 74, 75, 76]
TRPM7	MS	Upregulated in reactive astrocytes	Promotes glial scar formation via CSPGs production. Regulates macrophage or microglia activation.	FTY720 (FDA‐approved MS drug) that shows TRPM7 antagonism	[77, 78]
TRPM8	Migraine	Activated	Triggers CGRP secretion and COX‐2 expression via CaMKII‐dependent pathways. Drives neuroinflammatory events in the meninges via GFRα3.	*Trpm8* knockout TRPM8^+^‐neurons ablation TRPM8 antagonist (PBMC)	[79, 80, 81, 82]
	Ischemic stroke	Activated for protective functions	Exerts anti‐neuroinflammatory effects by suppressing glial activation and oxidative stress.	TRPM8 agonist (menthol)	[83]

Abbreviations: 2‐APB, 2‐aminoethoxydiphenyl borate; ACA, N‐(p‐amycinnamoyl) anthranilic acid; AD, Alzheimer's disease; ASD, autism spectrum disorder; BAFF, B‐cell activating factor; CBA, 4‐Chloro‐2‐(2‐(2‐chlorophenoxy) acetamido) benzoic acid; CCL2, chemokine (C‐C motif) ligand 2; CGRP, calcitonin gene‐related peptide; CSPGs, chondroitin sulfate proteoglycans, COX‐2, cyclooxygenase‐2; CXCL2, C‐X‐C motif chemokine ligand2; FDA, Food and Drug Administration; GFRα3, neurotrophic factor family receptor alpha‐3; IL‐, interleukin‐; iNOS, inducible nitric oxide synthase; MS, multiple sclerosis; NBA, 4‐Chloro‐2‐(2‐(naphthalene‐1‐yloxy) acetamido) benzoic acid; NG, nodose ganglion; NLRP3, nucleotide‐binding oligomerization domain‐like receptor pyrin domain containing 3; NOS2, nitric oxide synthase 2; PBMC, 1‐phenylethyl‐4‐(benzyloxy)‐3‐methoxybenzyl (2‐aminoethyl) carbamate; PD, Parkinson's disease; ROS, reactive oxygen species; TG, trigeminal ganglion; TLR4, Toll‐like Receptor 4; TNF‐α, tumor necrosis factor α.

**TABLE 5:** TRPV channels in neurological diseases.

TRPV channels	Disease	Expression or activity (change in disease)	Modulatory effects in neuroinflammation	TRP‐targeted approaches that show therapeutic effects	References
TRPV1	Neuropathic pain	Upregulated in microglia and DRG neurons	Increases the expression of inflammatory factors (IL‐1β, IL‐6, TNF‐α).	*Trpv1* deletion AM1710 (CB2 receptor agonists, suppresses TRPV1 expression) miR‐338‐3p overexpression (inhibits *Trpv1* expression) TRPV1 antagonists (SB366791, capsaicin) Capsaicin (reduces TRPV1 expression) CRAP (inhibits TRPV1 expression) Cinobufagin (inhibits TRPV1 upregulation) BoNTs (inhibits TRPV1 upregulation) EA (inhibits TRPV1 signaling) rTMS or TENS (reduces TRPV1 expression)	[33, 34, 84, 85, 86, 87, 88, 89, 90, 91, 92, 93]
	Migraine	Upregulated in TNC	Activates NLRP3 inflammasome and increases levels of serum TNF‐α, IL‐1β, and IL‐18.	XSZT (a traditional Chinese medicine formula, suppresses TRPV1 expression) AEA (a CB agonist, reduces TRVP1 expression)	[94, 95]
	Ischemic stroke	Both up‐ and downregulated	Leads to autophagy‐induced activation of the NLRP3 inflammasome. Increases the expression of TNF‐α, TLR2, and TLR4. Exerts antioxidant and anti‐inflammatory effects via p38 MAPK/TLRs (protective).	TRPV1 agonists (6‐gingerol, BCP, capsaicin) *Trpv1* knockout TRPV1 antagonist (capsazepine, AMG 9810) EA pretreatment (suppresses TRPV1 expression)	[31, 96, 97, 98, 99, 100]
	MS	Genetic polymorphisms: rs877610 (risk factor) and rs222747 (increased activity)	Protective: suppresses TNF‐α and IL‐6. Detrimental: exacerbates inflammation via Ca^2+^‐PP2A‐NLRP3 pathway.	*Trpv1* knockout TRPV1 agonists (capsaicin, RTX, SA13353)	[101, 102, 103, 104, 105]
	AD	Decreased	Reduces neuroinflammation by modulating microglial lipid metabolism via suppressing SREBP activity.	TRPV1 agonist (capsaicin) *Trpv1* knockout	[106, 107, 108, 109, 110, 111, 112, 113, 114]
	PD	Decreased in MPTP‐induced model Upregulated in 6‐OHDA‐induced model	Suppresses gliosis, NLRP3‐caspase1 signaling pathway, and release of neuroinflammatory cytokines.	TRPV1 agonist (capsaicin) AEA (a CB agonist, reduces TRVP1 expression) CBD (a phytocannabinoid, upregulates astrocytic TRPV1 expression) The Chinese medicinal herb LSC (increases expression of TRPV1) Targeted nanoparticles CS‐AT NPs (activates microglia surface TRPV1) EA treatment (suppresses TRPV1 expression) Rivastigmine (suppresses TRPV1 expression)	[37, 115, 116, 117, 118, 119, 120]
	ASD		Involved in pain and neuroinflammation associated with *SHANK3*‐related ASD.		[121]
	Epilepsy	Upregulated	1. Cooperates with IL‐1R1 to amplify proinflammatory cytokines (IL‐1β, TNF‐α, HMGB1). 2. Activates microglia via IL‐17A.	*Trpv1* knockout	[122, 123, 124, 125]
	Depression	Increased expression	Activates NLRP3 inflammasome pathway in microglia.	*Trpv1* knockout Metformin (suppresses TRPV1 expression) ACEA (a selective cannabinoid CB1 receptor agonist, suppresses TRPV1 expression)	[126, 127, 128]
	Anxiety	Activated		*Trpv1* knockout TRPV1 antagonist (AMG 9810)	[129, 130, 131, 132]
TRPV2	Ischemic stroke	Upregulated in astrocytes and microglia	Promotes the activation of glial cells (proliferation of astrocytes and polarization of microglia) and the release of inflammatory factors.	TRPV2 Inhibitor (Tranilast) TRPV2 negative allosteric modulator (plumbagin) CBD (reduces TRPV2 expression)	[133, 134, 135]
TRPV4	Neuropathic pain	Upregulated in spinal microglia	Increases the expressions of IL‐1β and IL‐6 in DRG. Promotes microglial activation and neuronal dysfunction via lipocalin‐2 release.	*Trpv4* knockout TRPV4 antagonists (RN‐1734, HC‐067047, GSK2193874)	[136, 137, 138]
	Ischemic stroke	Upregulated	Enhances proinflammatory cytokine release and activation of microglia.	TRPV4 antagonist (GSK2193874) EA (inhibits TRPV4 expression)	[139]
	MS	Upregulated by TNF‐α in brain endothelial cells	Disrupts BBB integrity (via claudin‐5 dysregulation) and amplifies T cell infiltration and inflammation.	*Trpv4* deletion	[140, 141]
	PD	Upregulated	Drives dopaminergic neuron loss via ER stress and inflammation.	*Trpv4* knockdown	[142]
	ASD	Upregulated in NAc		TRPV4 inhibitor (HC‐067047)	[143]
	Epilepsy	Upregulated in hippocampal astrocytes	Activates Microglia and astrocytes. Promotes NLRP3, ASC, and caspase‐1 expression Promotes release of IL‐1β, TNF‐α, and IL‐6.	TRPV4 antagonist (HC‐067047)	[36, 144, 145]
	Depression	Increased in the hippocampus	Increases astrocytes and microglia activation. Promotes CaMKII‐NLRP3 inflammasome expression.	Hippocampal *Trpv4* knockdown TRPV4 inhibitor (HC‐067047)	[146]

Abbreviations: 6‐OHDA, 6‐hydroxydopamine; ACEA, arachidonyl‐2′‐chloroethylamide; AD, Alzheimer's disease; AEA, anandamide; ASC, apoptosis‐related spotted protein; ASD, autism spectrum disorder; BBB, blood–brain barrier; BCP, beta‐caryophyllene; BoNTs, botulinum toxin; CaMKII, calcium/calmodulin‐dependent protein kinase II; Caspase‐1, cysteinyl aspartate‐specific protease‐1; CB, cannabinoid receptor; CBD, cannabidiol; CRAP, coumarins from Radix angelicae *pubescentis*; CS‐AT NPs, Cu₂₋ₓSe‐anti‐TRPV1 nanoparticles; DRG, dorsal root ganglion; EA, electroacupuncture; ER, endoplasmic reticulum; HMGB1, high mobility group box 1; IL‐, interleukin‐; LSC, Lignans of *Schisandra chinensis* (Turcz.) Baill; MAPK, mitogen‐activated protein kinase; MPTP, 1‐methyl‐4‐phenylpyridinium; MS, multiple sclerosis; NAc, nucleus accumbens; NLRP3, nucleotide‐binding oligomerization domain‐like receptor pyrin domain containing 3; PD, Parkinson's disease; PP2A, protein phosphatase 2A; rTMS, repetitive transcranial magnetic stimulation; RTX, resiniferatoxin; TENS, transcutaneous electrical nerve stimulation; TLR, Toll‐like receptors; TNC, trigeminal nucleus caudalis; TNF‐α, tumor necrosis factor α; XSZT, Xiongshao Zhitong granules.

### 4.1 Neuropathic Pain

Neuropathic pain arises from damage or dysfunction of the somatosensory nervous system, manifesting as spontaneous burning/shooting pain, allodynia (pain from nonpainful stimuli), and hyperalgesia (exaggerated pain response) [147]. Neuroinflammation is a central driver of neuropathic pain [148, 149]. Peripheral nerve injury activates immune responses, leading to peripheral and central sensitization. In the CNS, activated microglia release brain‐derived neurotrophic factor (BDNF) and IL‐6, and astrocytes sustain inflammation via connexin‐43 gap junctions and chemokine release, which further amplify pain signals. The TRP channel family participates in pain signaling, and the subtypes such as TRPA1, TRPM2, TRPV1, and TRPV4 are activated by a variety of physical and chemical stimuli and participate in the initial transmission and regulation of pain signals through neuroimmune signaling [11].

TRPA1, a polymodal nonselective cation channel, contributes to neuropathic pain. Both genetic deletion of *Trpa1* and pharmacological inhibitors of TRPA1 (HC‐030031 and A‐967079) reduce pain‐like behaviors in a trigeminal neuropathic pain mouse model, which may depend on reducing the expression of CCL2 in monocyte/macrophage [43]. However, another study showed that silencing of *Trpa1* in Schwann cells attenuates mechanical allodynia and neuroinflammation without affecting macrophage infiltration [44]. Whether TRPA1 modulates neuropathic pain via neuroinflammation still needs more investigations.

TRPM2 is highly expressed in immune cells, especially in macrophages and microglia, and its activation can exacerbate pro‐pain inflammatory responses in the peripheral and spinal cord [11]. *Trpm2* knockout shows analgesic effects in multiple pain mouse models [55]. In the models of carrageenan‐induced inflammatory pain and sciatic nerve injury–induced neuropathic pain, *Trpm2* knockout reduces mechanical and thermal hyperalgesia by decreased neutrophil infiltration and CXCL2 production, and suppressed spinal microglia activation [56]. A recent review claimed that TRPM2 and TLR4 interact with each other to regulate neuroinflammation, thereby affecting neuropathic pain [57].

TRPV1 plays a well‐established role in pain pathophysiology. During inflammation or tissue damage, TRPV1 becomes sensitized to enhance pain sensation. A marked increase of TRPV1 expression in DRG neurons is observed in several animal models, including pain models induced by chronic constriction injury (CCI), complete Freund's adjuvant (CFA), and spared nerve injury (SNI) [84, 85, 86, 87]. TRPV1 is also expressed in microglia, where it regulates pain by modulating microglial activity and neuron–microglia communication [88]. TRPV1 expression is regulated by various factors and signaling pathways in pain. In the CCI rat model, TRPV1 and N‐Terminal EF‐Hand Ca^2+^ Binding Protein 2 (NECAB2) together regulate the expression of inflammation‐related genes such as *Cox‐2*, *Tnf‐α*, and *Il‐6*, which contribute to nociceptive processing [86]. TRPV1 and β1 receptors co‐localize in non‐peptidergic sensory fibers and astrocytes, particularly in the spinal dorsal horn and DRG, often alongside IL‐1β in the unilateral partial sciatic nerve ligation (PSNL) rat model [89]. In the SNI model, TRPV1 is co‐expressed with cannabinoid receptor (CB) type‐1 and chemokines (CCL2 and CCL3) in pain‐modulating regions such as the hippocampus and thalamic nuclei [150]. TRPV1 interacts with endocannabinoids to orchestrate the pathophysiology of neuropathic pain [85]. Inhibition of the activity and expression of TRPV1 produces significant analgesic effects by inhibiting inflammation. For example, treatment with CB2 receptor agonists AM1710 reduces TRPV1 and CCL2 expression, accompanied by suppressing astrocyte activation in the spinal cord, thereby alleviating pain in CCI mice [90]. These anti‐allodynia effects of AM1710 are reversed by *Trpv1* knockout [90]. Another study showed that *Trpv1* deletion in the SNI model ameliorates mechanical and thermal hyperalgesia by reducing inflammatory factors including IL1‐β, IL‐3, IL‐6, IL‐12, IL‐17, TNF‐α, and IFN‐γ [91]. Overexpression of miR‐338‐3p, an upstream factor of TRPV1, suppresses TRPV1 expression and reduces neuropathic pain through downregulating inflammation [86]. In the PSNL model, TRPV1 antagonists (SB366791) reduce thermal and mechanical pain [89]. Capsaicin, lowering TRPV1 expression, reduces microglial inflammation and neuronal Ca^2+^ influx by inhibiting the TLR4/AKT/NF‐κB pathway, thereby relieving chronic pain in the CFA rat model [34]. In SNI models, coumarins from Radix angelicae *pubescentis* (CRAP) inhibit TRPV1 expression along with reduced proinflammatory cytokines (TNF‐α, IL‐1β, and IL‐6) and phosphorylation of ERK, producing an analgesic effect [33]. Cinobufagin relieves pain by inhibiting TRPV1 upregulation, reducing spinal astrocyte activation, and decreasing proinflammatory cytokines, thereby alleviating paclitaxel‐induced neuropathic pain [151]. The analgesic effect of botulinum toxin (BoNTs) depends on TRPV1 and substance P signaling, which mainly inhibits TRP‐associated neuroinflammation accompanied by downregulated pain‐promoting gene expression [92]. Moreover, nonpharmacological interventions, such as electroacupuncture (EA), repetitive transcranial magnetic stimulation (rTMS), and transcutaneous electrical nerve stimulation (TENS), have been developed to relieve neuropathic pain by targeting TRPV1 and neuroinflammation with different mechanisms. EA relieves neuropathic pain by inhibiting TRPV1 signaling through reducing inflammatory factors (IL‐1β, IL‐6, TNF‐α) and also suppresses astrocyte and microglia activation in multiple brain regions of SNI mice [91]. In CCI rats, both rTMS and TENS display analgesic effects, but rTMS outperforms TENS in reducing pain [93]. Specifically, rTMS applied to the prefrontal cortex (PFC) reduces TRPV1 expression in the spinal cord and lowers levels of IL‐1β, IL‐6, and TNF‐α in both the PFC and spinal cord. In contrast, TENS only decreases IL‐1β expression in these regions [93]. Therefore, downregulating TRPV1 channel activity may be a promising molecular target for treating neuropathic pain.

TRPV4 is expressed in DRG neurons and glial cells, as well as in immune cells in multiple brain regions [152]. Recent studies indicate that spinal activation of TRPV4 by GSK1016790A induces acute mechanical pain and increases the expression of IL‐1β and IL‐6 in the DRG [136, 137]. In a chronic compression of the DRG model, TRPV4 antagonists RN‐1734 and HC‐067047 relieve neuropathic pain and inflammation of astrocytes in the DRG [137, 138]. Similarly, blocking TRPV4 either genetically or with drugs such as GSK2193874 significantly reduces pain‐like behaviors in the SNI mouse model [136]. Mechanistically, upregulated TRPV4 expression is observed in spinal resident microglia and then promotes microglia activation, leading to functional and structural changes in excitatory spinal neurons by releasing lipocalin‐2 [136].

### 4.2 Migraine

Migraine is a disabling neurological disorder characterized by recurrent throbbing headaches accompanied by nausea, photophobia, and phonophobia, classified into episodic and chronic forms. While historically explained by vascular and neuronal hyperexcitability theories, emerging findings highlight neuroimmune dysregulation as a key contributor [153]. Peripheral and central immune activation, together with microglia and astrocyte activation in the thalamus and brainstem, drive the release of proinflammatory cytokines and amplify pain. The TRP channels are highly expressed in migraine‐associated trigeminal ganglion (TG) neurons and other pain‐related regions, where they drive the pathology of migraine and facilitate the release of CGRP, a key neuropeptide in migraine pathogenesis [154, 155, 156]. In particular, activated TRPA1, TRPM2, TRPM8, and TRPV1 channels participate in migraine progress through amplifying neuroinflammation.

TRPA1 channels, activated by oxidative stress products, depolarize TG neurons, thereby evoking CGRP release and neurogenic inflammation that ultimately precipitate migraine attacks [45]. Emerging evidence highlights the role of TRPA1 in glial activation, particularly in astrocytes, as a driver of neuroinflammatory processes contributing to migraine. In a nitroglycerin‐induced acute migraine mouse model, the selective TRPA1 antagonist ADM_12 significantly attenuates microglial and astrocytic activation within the trigeminal nucleus caudalis (TNC), and reduces satellite glial cell activation in the TG, consequently reversing hyperalgesia [46]. A recent review summarized that TRPA1 expression in pain syndromes is dynamically controlled through multiple epigenetic mechanisms, including DNA methylation, post‐translational histone modifications, and non‐coding RNA networks (microRNAs, long non‐coding RNAs, and circular RNAs) [47]. Additionally, TRPA1 modifies the enzymes for epigenetic modifications and non‐coding RNAs expression. TRPA1‐mediated neurogenic inflammation in migraine pathogenesis is regulated by these epigenetic modifications [47]. This bidirectional relationship between epigenetic regulation and neurogenic inflammation underscores TRPA1 as a potential therapeutic target for the intervention of migraine.

TRPM2 channel is also increased in the TG of nitroglycerin‐induced migraine mice [58]. TRPM2 blockers (N‐(p‐amycinnamoyl) anthranilic acid (ACA) and 2‐aminoethoxydiphenyl borate (2‐APB)) and *Trpm2* knockout reduce migraine‐like pain behaviors [59]. Both ACA and 2‐APB reduce ROS, apoptosis, cytosolic Ca^2+^ levels, and proinflammatory cytokine levels in TG [59].

TRMP8 has been identified as a migraine susceptibility gene [79, 80, 157]. *Trpm8* deletion or ablation of TRPM8‐expressed neurons abolishes nitroglycerin‐induced migraine‐like pain in mice, and the selective TRPM8 antagonist 1‐phenylethyl‐4‐(benzyloxy)‐3‐methoxybenzyl (2‐aminoethyl) carbamate (PBMC) also shows similar effects [81]. Intraperitoneal injections of CGRP, a migraine‐inducing method, do not cause migraine‐like nociceptive behaviors in *Trpm8* knockout mice [81], suggesting that TRPM8 plays an important role in the pathogenesis of migraine. Additionally, activation of cortical TRPM8 enhances KCl‐evoked spreading of depolarization‐induced cortical inflammation. In primary cultures of TG neurons, TRPM8 activation triggers the upregulation of CGRP and COX‐2 expression through a CaMKII‐dependent signaling pathway [82]. A recent study showed that TRMP8 is expressed in the meninges, and neuroinflammatory events in the meninges lead to migraine‐like pain via glial cell‐derived neurotrophic factor family receptor alpha‐3 (GFRα3)‐TRPM8 signal pathway [158]. The expression and colocalization of TRPM8 and TRPV1 in TG neurons are increased in a meningeal inflammation‐based migraine model [159].

TRPV1 also plays an important role in migraine pathogenesis [155], and a recent study confirms that TRPV1 is upregulated in TNC of the nitroglycerin‐induced migraine rat model [94]. The traditional Chinese medicine formula, Xiongshao Zhitong granules (XSZT), alleviates nitroglycerin‐induced migraine‐like behaviors by downregulating CGRP, TNF‐α, IL‐1β, and IL‐18 levels and reducing mast‐cell degranulation via suppression of the TRPV1/Ca^2+^/NLRP3 signaling pathway [94]. Similarly, the CB agonist anandamide (AEA) decreases the expression of TRPV1, nitric oxide synthase (nNOS), NF‐κB, and COX‐2 in the upper cervical spinal cord of the nitroglycerin‐induced migraine rat model [95], indicating TRPV1 may combine with the cannabinoid receptor in modulating inflammation in migraine pathogenesis. These findings suggest that TRP channels integrate multiple signaling pathways to drive neuroinflammatory processes in migraine.

### 4.3 Ischemic Stroke

Stroke, a leading cause of disability and mortality, is classified into two types: ischemic stroke (caused by blockage of cerebral blood flow) and hemorrhagic stroke (caused by vessel rupture). Following ischemic stroke, a cascade of pathological events unfolds, including glutamate excitotoxicity, Ca^2+^ overload, and oxidative stress [160]. Neuroimmune interactions critically modulate both injury and recovery processes after ischemic stroke [160, 161, 162]. Microglia are activated and shift to the proinflammatory phenotype within minutes of ischemia, thereby promoting proinflammatory cytokines such as TNF‐α and IL‐1β release that amplify BBB disruption and accelerate neuronal death. Astrocytes subsequently contribute to glial scar formation that limits damage expansion but may inhibit axonal regeneration. In the later phase, microglia transition to an anti‐inflammatory phenotype to promote tissue repair by clearing debris, secreting neurotrophic factors (e.g., BDNF). Different TRPs show distinct roles in ischemic stroke. Three TRP channel members (TRPC1, TRPC6, and TPRV1) exhibit dual roles [18]. TRPM2, TRPV2, and TRPV4 exacerbate ischemic stroke, while TRPM8 plays a protective role.

#### 4.3.1 TRPC1, TRPC6, and TPRV1 Play Dual Roles

TRPC1 is upregulated and SUMOylation in microglia in middle cerebral artery occlusion reperfusion (MCAO/R) mice and oxygen and glucose deprivation/reoxygenation (OGD/R) cell model [16, 49]. Microglia conditional *Trpc1* knockdown reduces infarct volumes and alleviates neurological deficits by decreasing CCL5 and CCL2 secretion [16], and SUMOylated TRPC1 also activates the NLRP3 signaling pathway, promoting the production of CCLs and CXCLs in microglia in MCAO/R mice [49]. However, a study oppositely found that TRPC1 shows a beneficial effect for stroke in mice [50].

TRPC6 generally confers a neuroprotective role in ischemic stroke by preventing neuronal death [52]. The proinflammatory cytokine IL‐17A acts as upstream to promote TRPC6 degradation, exacerbating brain injury [53]. In MCAO/R mice and OGD/R cultured astrocytes, TRPC6 expression is decreased. Treatment with the selective TRPC6 agonist HYP9 increases the number of astrocytes and reduces infarct size and levels of IL‐6 and IL‐1β, whereas the TRPC antagonist SKF96365 exacerbates the damage [39]. HYP9 treatment or *Trpc6* overexpression in OGD/R astrocytes attenuates apoptosis, inflammatory responses, and NF‐κB phosphorylation [39]. However, studies demonstrated a detrimental effect of TRPC6 in ischemic stroke. Increased TRPC6 expression is observed in the cortical neurons after OGD/R, and *Trpc6* knockout protects mice against ischemic stroke [54, 163].

TRPV1 also exhibits a dual role in ischemic stroke, with both agonists and antagonists demonstrating neuroprotective potential in specific contexts [164]. TRPV1 channel activators, beta‐caryophyllene (BCP) or 6‐gingerol, exhibit neuroprotective effects in cerebral ischemia rats through reducing neuroinflammation [96, 97]. BCP pretreatment upregulates TRPV1 expression in activated astrocytes and ameliorates early neuroinflammation in a bilateral common carotid artery occlusion followed by reperfusion (BCCAO/R) rat model [97]. Moreover, 6‐gingerol treatment reduces cerebral infarct volume, brain edema, and morphological damage, and improves neurological scores in MCAO/R rats [96]. These protective effects of 6‐gingerol are attributed to inhibiting apoptosis and autophagy‐induced NLRP3 inflammasome activation by disrupting the interaction between TRPV1 and Fas‐associated factor 1 (FAF1), which is abolished by *Trpv1* knockdown [96]. Similarly, the TRPV1 agonist capsaicin suppresses the expression of NLRP3, cysteinyl aspartate‐specific protease‐1 (caspase‐1), and IL‐1β by anti‐autophagy in microglia after OGD/R, whereas the TRPV1 antagonist 5′‐iodoresiniferatoxin (iRTX) exhibits the opposite effect [165], suggesting that TRPV1 modulates autophagy‐mediated NLRP3 inflammatory effects may be a key neuroprotective target in ischemic stroke. Conversely, knockout of *Trpv1* or pharmacological inhibition with capsazepine or AMG9810 alleviates neurological and motor deficits in the MCAO/R model [98, 99, 100]. The neuroprotective effects of AMG9810 may be due to increased anti‐inflammatory cytokine (IL‐10) and reduced inflammatory factors (TNF‐α, TLR2, and TLR4) [99, 100]. Another study demonstrated that EA pretreatment enhances neurological scores and reduces infarct volumes in MCAO/R rats through anti‐inflammatory mechanisms by suppressing TRPV1 expression and p38 MAPK activation [31]. Thus, the above results indicate that either insufficient or excessive TRPV1, TRPC6, and TRPC1 signaling can aggravate brain injury in ischemic stroke.

#### 4.3.2 TRPM2, TRPV2, and TRPV4 Play Detrimental Roles

TRPM2, TRPV2, and TRPV4 exacerbate neuroinflammation and worsen outcomes in ischemic stroke. Increased expression or activation of TRPM2, TRPV2, and TRPV4 was observed in stroke [60, 133, 139]. An in vitro study in PC12 cells demonstrated that knockdown of *Trpm2* shows neuroprotection after OGD/R by reducing apoptosis, Ca^2+^ levels, ROS production, and NLRP3‐mediated inflammasome [61].

Following OGD/R in rat hippocampal slices, the TRPV2 expression is increased in microglia. While cannabidiol (CBD), a rigorously studied phytocannabinoid, reduces this upregulation and the number of TRPV2‐positive phagocytic microglia [134]. In the MCAO/R model, a specific TRPV2 negative allosteric modulator plumbagin reduces cerebral infarction and neurological deficit by anti‐inflammatory polarization of microglia, evidenced by increasing transcript levels of anti‐inflammatory factors *Arg1*, *Il‐10*, and *transforming growth factor β* (*Tgf‐β*), while decreasing proinflammatory factors *Tnf‐α*, *Il‐1β*, and *Il‐6* [135]. In the OGD/R‐induced astrocytes model, the TRPV2 expression is upregulated, and the TRPV2 inhibitor tranilast restores the proliferation of astrocytes via the JNK‐NGF pathway [133].

TRPV4 is upregulated after ischemic brain injury, and its activation promotes the activation of microglia and the release of proinflammatory cytokines. The TRPV4 antagonist GSK2193874 or EA‐mediated TRPV4 suppression reduces cerebral infarction and neurological dysfunction in the MCAO/R model by suppressing the activation and proinflammatory polarization of microglia and the mRNA levels of *Tnf‐α*, *Il‐6*, and *ccl2* [139]. Therefore, inhibition of TRPM2, TRPV2, and TRPV4 channels emerges as promising anti‐neuroinflammatory targets for stroke treatment.

#### 4.3.3 TRPM8 Plays a Protective Role

In contrast, TRPM8 activation shows neuroprotection in ischemic stroke. Topical application of menthol to all paws' derma, a Food and Drug Administration (FDA) approved TRPM8 agonist, decreases infarct volume, improves sensorimotor function, and suppresses glial activation, immune cell infiltration, and oxidative stress in MCAO/R mice [83]. These beneficial effects can be reversed by the TRPM8 antagonist AMTB or lidocaine, or by *Trpm8* knockout, highlighting TRPM8's therapeutic potential in ischemic stroke treatment.

### 4.4 Multiple Sclerosis (MS)

MS is a chronic autoimmune disorder of the CNS characterized by demyelination, axonal damage, and neuroinflammation [166]. Clinical manifestations include fatigue, motor weakness, sensory deficits, and cognitive impairment, driven by immune‐mediated attacks on myelin sheaths and oligodendrocytes [166]. Neuroimmune dysregulation, both in the peripheral and in the CNS, has a great impact on MS pathogenesis. In the CNS, microglia and astrocytes are activated by proinflammatory signals, releasing ROS and excitotoxins, which exacerbate demyelination and neuronal loss under MS [167]. TRP channels are emerging as key regulators of MS pathogenesis, controlling neuroinflammation, BBB integrity, glial reactivity, and remyelination. Among them, TRPV1 exerts context‐dependent dual actions within the evolving MS lesion, while TRPM2, TRPM4, TRPM7, and TRPV4 consistently drive detrimental inflammatory responses.

#### 4.4.1 TRPV1 Plays a Dual Role

TRPV1 channel acts as a critical modifier and may play a dual role during MS. Several studies have indicated that the missenses of *TRPV1* single‐nucleotide polymorphism (SNP) are closely associated with the progression of MS [101, 102, 103]. TRPV1 SNP rs877610 is a risk factor for MS [101], whereas rs222747 increases the expression and activity of TRPV1 and may have good benefit to MS patients due to the lower levels of TNF‐α in cerebrospinal fluid (CSF) [102]. In experimental autoimmune encephalomyelitis (EAE) mice, a classical animal model for MS, TRPV1 channel protects synaptic damage in the early phase of EAE and enhances IL‐1β‐induced deficits in the chronic stages [104]. On one hand, *Trpv1* deficiency alleviates the damage in EAE mice by reducing NLRP3 inflammasome‐mediated neuroinflammation of microglia via inhibiting Ca^2+^‐protein phosphatase 2A (PP2A) signaling [101, 102, 105]. On the other hand, TRPV1 agonists, such as capsaicin, resiniferatoxin (RTX), and SA13353, significantly attenuate cytokine levels including TNF‐α and IL‐6 in both in vitro and in vivo studies [102, 168]. However, how these agonists modulate the cytokine release in the EAE model has not been explored.

#### 4.4.2 TRPM2, TRPM4, TRPM7, and TRPV4 Play Detrimental Roles

Activation of TRPM2, TRPM4, and TRPM7 channels shows detrimental effects during MS. TRPM2, highly expressed in the brain and immune cells, plays an important role in EAE mice. Genetic deletion of *Trpm2* or pharmacological inhibition of TRPM2 with miconazole, an FDA‐approved antifungal drug, alleviates neurological and pathological impairment of EAE pathology by downregulating microglial activation and inhibiting proinflammatory cytokines production in MS mouse models [62, 63]. Further results indicate that these protective effects in *Trpm2* knockout mice were via inhibiting the NLRP3 inflammasome activation [62].

Upregulated TRPM4 in EAE models is positively correlated with disease progression [72, 73]. Reducing the expression and activation of TRPM4 by genetic deletion or the TRPM4 inhibitors, such as 9‐phenantrol, glibenclamide, 4‐Chloro‐2‐(2‐(2‐chlorophenoxy) acetamido) benzoic acid (CBA), and 4‐Chloro‐2‐(2‐(naphthalene‐1‐yloxy) acetamido) benzoic acid (NBA), shows a promising protective effect in MS [72, 74]. Further studies indicate that these effects may be due to downregulated sulfonylurea receptor 1 (Sur1)–TRPM4 pathways in astrocytes, thereby reducing the levels of several proinflammatory mediators such as TNF‐α, IFN‐γ, IL‐17, B‐cell activating factor (BAFF), CCL2, and nitric oxide synthase 2 (NOS2) [75, 76].

TRPM7 is abundantly present in reactive astrocytes at MS lesion sites, and boosts glial scar formation and restrains neuronal synaptic growth by elevating chondroitin sulfate proteoglycans (CSPGs) production [77]. Moreover, FTY720, an FDA‐approved MS drug, inhibits TRPM7 function, and VPC01091.4, an analog of FTY720, reduces cytokine release from inflammatory‐activated macrophage/microglia by inhibiting TRPM7 currents [78]. The above findings suggest that the activation of TRPM2, TRPM4, and TRPM7 channels aggravates neuroinflammation and worsens MS‐related pathology. Therefore, targeted strategies for lowering the expression or activity of these TRPM channels may offer a potential therapeutic approach for MS.

Additionally, activated TRPV4 may have bad effects on MS pathophysiology. TRPV4 exhibits a distinct spatial expression pattern in MS, characterized by elevated endothelial expression specifically in regions adjacent to mixed active/inactive lesions within MS brain tissues, which correlates with microglial activation [140]. Mechanistic investigations reveal that upregulated brain endothelial TRPV4 expression, derived from microglial TNF‐α, leads to claudin‐5 dysregulation, thereby disrupting BBB integrity and amplifying inflammation such as T cell infiltration and inflammatory mediators release [140]. However, a recent study showed that global or microglia‐specific deletion of *Trpv4* increases microglial phagocytic activity, but has no impact on disease severity and myelin damage extent in EAE and cuprozone‐induced demyelination MS models [141]. Therefore, how TRPV4 participates in the pathology of MS needs further studies.

### 4.5 Alzheimer's Disease (AD)

AD, the most common neurodegenerative disorder, is characterized by amyloid‐β (Aβ) plaques and hyperphosphorylated tau‐induced neurofibrillary tangles (NFTs) in the brain, which lead to neuronal death and memory loss. Microglia exhibit a dual role in AD. They clear Aβ in the early stage, but chronic activation shifts them to a proinflammatory state, releasing cytokines that exacerbate neurodegeneration [169]. Astrocytes, activated by Aβ, contribute to neuroinflammation by upregulating the complement cascade and promoting excitotoxicity by impairing glutamate clearance [170]. Microglia and astrocytes' dysfunction highlights the critical role of neuroinflammation in AD progression [171, 172]. TRP‐channel‐mediated glial dysfunction is emerging as an important pathogenic axis in AD. TRPV1 exerts a bidirectional influence, whereas TRPA1 and TRPM2 drive deleterious signaling.

#### 4.5.1 TRPV1 Plays a Dual Role

TRPV1 may play a pleiotropic role during AD pathology. On one hand, several studies have revealed that TRPV1 shows a protective role in AD by modulating neuroinflammation [106, 107, 108]. Decreased TRPV1 expression is observed in APP23/PS45 double transgenic AD model mice [109]. Pharmacological activation of TRPV1 by capsaicin rescues Aβ‐induced microglial immune dysfunction, including phagocytic activity, and reverses memory deficits in the AD mice model [108, 110]. Recently, studies have shown that TRPV1 reduces neuroinflammation in AD by modulating microglial lipid metabolism via suppressing sterol regulatory element‐binding protein (SREBP) activity [111, 112]. The microglia in 3xTg mice, a transgenic mouse model of AD carrying three AD‐related mutant genes, exhibit significant lipid droplet accumulation, along with a marked upregulation of pyruvate kinase M2 (PKM2) and SREBP1. Pharmacological activation of TRPV1 by capsaicin restores transcriptional alterations of gene sets involved in lipid metabolism, innate immunity, and phagocytic function disorders in these microglia through inhibiting PKM2 dimerization and SREBP1 activation [112]. Microglia derived from induced pluripotent stem cells of *APOE* ε4 (a genetic risk factor for sporadic AD)‐bearing AD patients exhibit overactivation and overproduce cholesterol, boosting major histocompatibility complex II (MHC II)‐dependent antigen presentation, which can be reversed by the TRPV1 agonist capsaicin [111]. Moreover, in the *APOE* ε4‐related tauopathy mouse model, microglial TRPV1 loss worsens glial inflammation, T cell infiltration, and memory impairments, whereas capsaicin‐mediated TRPV1‐Ca^2+^ signaling blocks SREBP2‐driven cholesterol biosynthesis and effectively suppresses the excessive neuroinflammation and neurodegeneration [111]. On the other hand, evidence shows that *Trpv1* knockout in 3xTg mice restores memory loss and decreases Aβ and Tau burden by reducing Ca^2+^ influx via BDNF/CREB signaling in the hippocampus [113, 114].

#### 4.5.2 TRPA1 and TRPM2 Play Detrimental Roles

Aβ‐induced elevation of TRPA1‐Ca^2+^ signaling is observed in astrocytes of APP/PS1 mice, a widely used double transgenic AD mouse model [38]. Knockout of *Trpa1* or blockade of TRPA1‐channel function by the selective antagonist HC‐030031 restores cognition and reduces Aβ plaque deposition and proinflammatory cytokine (IL‐1β, IL‐6, and IL‐4) release in AD mice through the Ca^2+^‐PP2B/NF‐κB signaling pathway [38].

Similarly, decreased glutathione or ROS production activates the TRPM2 channel by increasing Ca^2+^ influx in aging neurons and glial cells [64]. Knockout of *Tprm2* in APP/PS1 mice reduces microglial activation and rescues spatial memory deficits [65]. Another study showed that ROS‐induced NLRP3 inflammasome production depends on TRPM2 activation in Aβ‐induced microglial cells [66]. These results indicate that modulating the TRPM2 channel activity in glia may be a potential target in the intervention of AD.

### 4.6 Parkinson's Disease (PD)

PD is a progressive neurodegenerative disorder marked by motor symptoms such as resting tremor, bradykinesia, rigidity, and postural instability, as well as non‐motor features like anosmia, constipation, sleep disturbances, and cognitive decline. Pathologically, PD is defined by the selective loss of dopaminergic neurons in the substantia nigra (SN) pars compacta and the accumulation of protein aggregates known as Lewy bodies, which are primarily composed of misfolded α‐synuclein [173]. Growing evidence underscores the critical role of neuroimmune dysregulation in driving the pathogenesis of PD [174]. Several TRP channels (TRPM2 and TRPV4 for detriment, TRPV1 for dual role) participate in the progress of PD via modulating neuroimmune.

#### 4.6.1 TRPV1 Plays a Dual Role

TRPV1 exhibits a paradoxical role in PD pathology. Decreased TRPV1 expression is observed in 1‐methyl‐4‐phenylpyridinium (MPTP)–induced PD mouse models [37]. Activation of TRPV1 by capsaicin shows protective effects on nigrostriatal DA neurons, which include reduced gliosis, decreased protein expressions of the NLRP3‐caspase1 signaling pathway, suppressed neuroinflammatory cytokine release, and improved behaviors in MPTP mice, and such effects are reversed by TRPV1 antagonists capsazepine and iRTX [115, 116, 117]. Besides, treatment with AEA (an agonist for CB and TRPV1) shows similar protective effects to capsaicin treatment in MPTP‐induced mice, and the benefits of capsaicin are abolished by CB1/2 antagonists (AM251 and AM630) [117]. Another study showed that CBD improves motor behaviors and alleviates nigrostriatal degeneration in PD rats by upregulating astrocytic TRPV1 expression and reducing neuroinflammatory markers in the substantia nigra [118]. These results indicate that the crosstalk between CB and TRPV1 plays a vital role in PD pathology. Moreover, lignans of *Schisandra chinensis* (Turcz.) Baill (LSC), a Chinese medicinal herb, inhibit PD progression by mitigating neuroinflammation‐autophagy through restored expression of TRPV1 and thus upregulating AMPK and downregulating the NLRP3‐caspase1 signaling pathway [37]. A recent study showed that Cu₂₋ₓSe‐anti‐TRPV1 nanoparticles (CS‐AT NPs), a novel nanoparticle synthesized using copper‐deficient selenide with conjugated TRPV1 antibody, selectively targets to activate microglial surface TRPV1 under laser. This process, which subsequently induces the Ca^2+^/CaMKK2/AMPK/mTOR signaling pathway, and enhances microglial phagocytosis and degradation of the α‐synuclein (α‐syn) protein [119]. However, another study showed that TRPV1 is upregulated in the hippocampus and prefrontal cortex of 6‐hydroxydopamine (6‐OHDA)–treated mice, another PD animal model, and both EA treatment and rivastigmine reduce this upregulation, normalize plasma levels of inflammatory cytokines (e.g., IL‐1β, TNF‐α, IL‐6), and improve cognitive performance [120].

#### 4.6.2 TRPM2 and TRPV4 Play Detrimental Roles

Both TRPM2 and TRPV4 are upregulated in PD, which amplifies the neuroinflammation. TRPM2 is activated in the 1‐methyl‐4‐phenylpyridinium (MPTP)‐induced PD mouse model, and deletion of *Trpm2* in microglia or blockade of TRPM2 channel activity using ACA and 2‐APB suppresses inflammatory cytokines (IL‐1β, TNF‐α, IL‐6) responses and reduces MPTP‐induced damage [67]. AG490, a TRPM2 inhibitor, restores motor behavior by reverting the morphology of microglia and astrocytes to normal states, with normalization of mRNA expressions of proinflammatory (iNOS and CD86) and anti‐inflammatory mediators (Arginase1) in the striatum of the 6‐OHDA‐induced PD mouse model [68].

Similarly, TRPV4 expression is upregulated in the SN of MPTP‐induced PD mice, and overexpressing TRPV4 leads to movement disorders in mice [142]. Knockdown of *Trpv4* in the PD mouse model alleviates the movement deficits and the loss of dopamine (DA) neurons in the SN by attenuating endoplasmic reticulum (ER) stress and inflammation [142].

### 4.7 Autism Spectrum Disorder (ASD)

ASD, a neurodevelopmental disorder, characterized by social deficit and repetitive behaviors. Emerging evidence suggests that genetic and environmental factors both contribute to the risk of ASD. Recently, neuroimmune dysregulation and abnormal inflammatory responses have been extensively studied in ASD [175]. Activation of TRPV4 promotes ASD pathology, whereas TRPM2 and TRPV1 may show the opposite effect.

#### 4.7.1 TRPV4 Plays a Detrimental Role

A recent study showed that nucleus accumbens (NAc)‐specific neonatal downregulation of *Shank3*, a candidate gene of ASD, in mice results in abnormal social behaviors and upregulated TRPV4 expression [143]. Lipopolysaccharide (LPS)‐treated *Shank3* heterozygous mice exhibit social deficits, upregulated inflammatory markers IL‐1β and TNF‐α expression, and elevated TRPV4 expression in the striatum. Acute TRPV4 inhibitor HC‐067047 injection in NAc ameliorates sociability deficits in these mice [143].

#### 4.7.2 TRPM2 and TRPV1 Play Protective Roles

A robust reduction of *Trpm2* mRNA level, but not *Trpv1*, *Trpv4*, and *Trpm8* levels, is observed in the nodose ganglion (NG) of mice with knockout of *Shank3* [69]. In a systemic inflammation model induced by LPS, *Shank3*‐deficient mice exhibit more severe hypothermia and higher serum IL‐6 levels, and specific knockout of *Trpm2* in vagal sensory neurons of NG also induces similar phenotypes [69]. Besides, a review speculates that oxytocin release, a critical modulator in social behaviors, is dependent on the interaction between CD38 and TRPM2‐related Ca^2+^ influx [176]. This implicates a new role of TRPM2 as a potential target for therapeutic investigation of social dysfunction in ASD.

Moreover, TRPV1 is expressed and interacts with SHANK3 in DRG sensory neurons, and the knockdown of *SHANK3* in human DRG neurons disrupts TRPV1 function [121]. *Shank3* knockout mice display decreased heat hyperalgesia after inflammation or nerve injury, as well as in capsaicin‐induced pain, which may be attributed to diminished TRPV1 currents in the DRG neurons [121]. These results suggest that TRPV1 may be involved in the dysregulation of pain perception associated with *SHANK3*‐related ASD.

Besides, TRPC3, TRPC6, and TRPM7, important activators of neuroinflammation, have been implicated in ASD pathology [177]. However, how these TRP channel members are involved in the neuroinflammatory pathology that leads to behavioral deficits in ASD still requires further investigation.

### 4.8 Epilepsy

Epilepsy is a chronic neurological disease characterized by recurrent seizures, which are episodes of abnormal electrical activity in the brain. These seizures can manifest as involuntary movements, loss of consciousness, impaired awareness, and in some cases, loss of bowel or bladder control [178]. A hallmark of epilepsy is the association with increased production of inflammatory cytokines, which contribute to neuroinflammation, progressive brain damage, and the propagation of seizure activity [178, 179]. This interplay between inflammation and seizure activity underscores the complex pathophysiology of the condition. Studies have shown that activation of several TRP channels, including TRPA1, TRPC3, TRPM2, TRPV1, and TRPV4, modulates neuroinflammation and promotes seizures.

Activation of TRPA1 by trans‐cinnamaldehyde (TCA), an agonist of TRPA1, induces seizure activity in rats [180]. Additionally, upregulated TRPA1 expression is observed in the hippocampus of kainic acid– (KA‐) induced rat epilepsy model, and EA treatment reverses hippocampal neurons overexcitation by reducing TRPA1 and pERK1/2 (an inflammatory mediator) levels [48]. These results indicate that TRPA1‐mediated neuroinflammation may be closely related to epilepsy.

Elevated TRPC3 is observed in the seizure rat model, and a specific TRPC3 inhibitor Pyr3 attenuated the susceptibility and severity of seizures with a reduction of neuronal death and neuroinflammation in hippocampal neurons [51].

Moreover, the expression of TRPM2 is increased in the hippocampus after epilepsy occurred and *Trpm2* knockout alleviated the level of neuroinflammation and glial cell activation and ameliorated the epileptic‐related psychological disorders [70]. Further study showed that *Trpm2* knockout in microglia, but not in astrocytes, attenuates epileptogenesis and suppresses glial activation and neuroinflammation by upregulating autophagy [71].

Upregulated TRPV1 is found in both brain samples from epileptic patients and animal models of seizures [122]. TRPV1 activation by capsaicin promotes repetitive febrile seizures via elevated proinflammatory cytokines, such as IL‐1β, IL‐6, TNF‐α, and high mobility group box 1 (HMGB1), and deletion of *Trpv1* decreases seizure susceptibility [123]. TRPV1 interacts with IL‐1R1 receptor and upregulates the expression of IL‐1R1 receptor, thus promoting neuroinflammation during the febrile seizures [123]. Additionally, upregulated TRPV1 in proinflammatory Th17 cells promotes neuroinflammation by activating microglia via IL‐17A in a complex febrile seizures mouse model, and knockout of *Trpv1* attenuates Th17 cell differentiation and reduces seizure susceptibility [124]. A recent study showed that hypoxia‐ischemia induced TRPV1 upregulation in astrocytes promotes the occurrence of epilepsy by increasing the proinflammatory cytokines release, such as TNF‐α, IL‐6, IL‐1β, and iNOS [125]. Moreover, pretreatment with CBD attenuates both seizure activity and IL‐6 levels in the prefrontal cortex of pentylenetetrazole‐induced seizure mice, which are abolished by co‐administration of the CB1, CB2, or TRPV1 antagonists [181]. Mechanistically, CBD inhibits the seizure‐induced transition of microglia to a proinflammatory phenotype [182].

TRPV4 is upregulated in hippocampal astrocytes from human temporal lobe epilepsy patients [36, 144]. Activation of TRPV4 by the agonist GSK1016790A promotes microglia and astrocyte activation and enhances the neuroinflammatory response, suggested by the increase of NLRP3, apoptosis‐related spotted protein (ASC), and caspase‐1 expression and the release of proinflammatory cytokines (IL‐1β, TNF‐α, and IL‐6) [145]. Hippocampal release of IL‐1β, TNF‐α, and IL‐6 induced by GSK1016790A is inhibited using the TLR4 antagonist TAK‐242 or the NF‐κB pathway inhibitor BAY 11–7082 [35], which indicates that TRPV4 promotes neuroinflammation via activating TLR4/NF‐κB signaling. Inhibition of TRPV4 by the specific antagonist HC‐067047 mitigates seizures by alleviating the activation of astrocytes, the NLRP3 inflammasome, and cytokine production in seizure model mice [36, 145]. Mechanistically, HC‐067047 reverses seizures by targeting astrocytic TRPV4‐related neuroinflammation through the TRPV4/Ca^2+^/YAP/STAT3 signaling pathway [36]. These results indicate that upregulation of multiple TRP channels including TRPA1, TRPC3, TRPM2, TRPV1, and TRPV4 activates glia and promotes epilepsy. Therefore, small molecular drugs that inhibit these TRP channels hold great potential to be developed as targeted therapeutic strategies for epilepsy.

### 4.9 Psychiatric Disorders

Anxiety and depression are prevalent psychiatric disorders characterized by persistent emotional dysregulation. Anxiety manifests as excessive fear, hypervigilance, and avoidance, while depression is characterized by a persistent low mood or a significant loss of interest and pleasure in activities for long periods of time. Anxiety and depression result from a complex interaction of social, psychological, and biological factors, which have a close relationship with and are affected by physical health. Several studies indicate that TRPV1 and TRPV4 channel‐related neuroinflammation participates in anxiety and depression.

TRPV1 is involved in chronic pain‐related anxiety, behavioral, and associated inflammation [183]. The TRPV1 agonist capsaicin induced anxiety‐related behaviors, while the TRPV1 antagonist AMG 9810 showed anxiolytic‐like effects [129]. Moreover, several studies showed that *Trpv1* deletion reduces anxiety‐related behavior in rodent models [130, 131, 132]. TRPV1‐deleted mice also exhibited significantly reduced depression‐like behaviors in the forced swim test [126]. TRPV1 signaling is involved in pain‐induced and obesity‐related depression [184, 185]. Another study showed that increased TRPV1 expression activates the NLRP3 inflammasome pathway in microglia, promoting the allergic rhinitis‐associated depressive‐like behavior in mice. Metformin, a most commonly used drug for type 2 diabetes, reversed these symptoms by reducing TRPV1‐NLRP3 pathway expression in the hypothalamus [127]. Moreover, arachidonyl‐2′‐chloroethylamide (ACEA), a selective CB1 receptor agonist, suppresses TRPV1 expression and reverses the corticosterone‐induced depressive behaviors in mice, accompanied by reduced proinflammatory cytokine (IL‐6, TNF‐α, and IL‐1β) release and increased anti‐inflammatory IL‐10 levels in the hippocampus [128]. These results suggest that the function of TRPV1 is related to emotion regulation, and TRPV1 may be a candidate target for new antidepressant and antianxiety drugs.

Besides, LPS‐induced depression in mice is associated with increased TRPV4 in the hippocampus [146]. The TRPV4 inhibitor HC‐067047 or hippocampal *Trpv4* knockdown alleviates depression‐like behaviors by reducing astrocyte and microglia activation, with suppressed CaMKII‐NLRP3 inflammasome expression and serum IL‐6, TNF‐α, and IL‐1β levels, and increased neurogenesis in the hippocampus [146]. Thus, TRPV4 plays a key role in LPS‐induced depression‐like behaviors.

## 5 TRP Channel‐Targeted Therapies for Managing Neuroinflammation in Neurological Disorders

TRP channels contribute to neuroinflammatory responses through mechanisms involving Ca^2+^‐signaling, oxidative stress, and immune cell activation, thereby influencing disease progression and severity. Targeting TRP channels may offer novel therapeutic strategies for managing neuroinflammatory components in several neurological disorders. There are several FDA‐approved drugs targeted to TRP channels, including NGX‐4010 [186], menthol [187], RTX [188], miconazole [63], and FTY720 [78]. For example, TRPV1 agonist NGX‐4010 shows efficacy in relieving neuropathic pain in clinical trials [189], but its modulation of inflammation has not been addressed. Menthol improves the outcome of ischemic stroke in mice by targeted activation of TRPM8 [83], and these effects are currently being investigated in a clinical trial (NCT05877079). RTX, a highly potent TRPV1 agonist, is used in pain treatment, including bone cancer–derived pain [188] and inflammatory hyperalgesia [190]. RTX, miconazole, and FTY720 show therapeutic potency with anti‐proinflammatory effects in MS mouse models [63, 78, 102], but whether these drugs have potential for MS patient treatment still needs investigation. Therefore, more preclinical and clinical studies are needed to verify the therapeutic effects of these FDA‐approved drugs for different neurological disorders.

Besides, many TRP channel‐targeting small molecules that exhibit neuroinflammation modulatory effects are applied for the treatment of several neurological diseases. A967079 is a selective TRPA1 antagonist that attenuates CGRP levels (a key mediator of neuroinflammation) and pathological pain signaling [191]. JNJ‐28583113, a highly brain‐penetrable TRPM2 antagonist, suppresses the phosphorylation of glycogen synthase kinase‐3 (GSK3) α and GSK3β, thereby protecting cells against oxidative stress‐induced apoptosis and mitigating neuroinflammation by reducing the production and release of proinflammatory cytokines [192]. Therefore, JNJ‐28583113 may have great potential for the treatment of neurological disorders including ischemic stroke, MS, AD, and PD. AMG 9810 acts as a competitive TRPV1 antagonist by blocking capsaicin‐induced receptor activation. This mechanism effectively reverses hyperalgesia in animal models of inflammatory pain [193]. Capsaicin, an agonist of TRPV1, reduces neuroinflammation in ischemic stroke, MS, AD, and PD, which improves the outcomes of animal models of these diseases (Table 5). The safety of capsaicin has been tested clinically (NCT01621685), and its therapeutic effects are investigated in several clinical trials for pain treatment (NCT02441660) and stroke (NCT04052178). Therefore, these TRP channel‐targeting small molecules have good prospects in the treatment of neurological diseases.

## 6 Conclusion and Future Perspectives

TRP channels constitute a structurally and functionally diverse family of ion channels pivotal to both physiological processes and pathological conditions. Within complex mechanisms, this ion channel family fulfills multiple roles, including the regulation of Ca^2+^ homeostasis, modulation of cell proliferation, mediation of sensory signaling, and participation in inflammatory responses. In this review, we focus on the role of several TRP channels, including TRPA1, TRPC1, TRPC3, TRPC6, TRPM2, TRPM4, TRPM7, TRPM8, TRPV1, TRPV2, and TRPV4, in neuroinflammation under different neurological disorders including neuropathic pain, migraine, stroke, MS, AD, PD, ASD, epilepsy, and psychiatric disorders (Figure 3). These channels exhibit structural diversity, are sensitive to various stimuli, and are widely expressed in neurons and glial cells. They play important roles in neurological disorders, either exacerbating or mitigating disease conditions. Moreover, most TRP channel members are activated or upregulated to drive NLRP3 inflammasome activity and promote the release of proinflammatory cytokines (e.g., IL‐1β, TNF‐α, IL‐6), consequently exacerbating diseases (Tables 2, 3, 4, 5). TRPA1 is upregulated in pain pathophysiology (e.g., neuropathic pain and migraine), neurodegenerative diseases (e.g., AD), and epilepsy, where it amplifies neuroinflammation and worsens clinical symptoms (Table 2). Pharmacological or genetic suppression of TRPA1 activity attenuates these pathological changes (Table 2). Although TRPA1 is expressed in both neurons and astrocytes, the specific cell type in which its activation is most critical for disease progression, and the underlying mechanisms, remain to be defined and require further investigation. Similarly, TRPM2 is activated by ROS and drives the pathology of most neurological disorders, except ASD, where TRPM2 expression is downregulated (Table 4). This downregulation in ASD mouse models suggests that TRPM2 is required to preserve neuronal and glial homeostasis under physiological conditions. Pathological ROS accumulation, conversely, drives TRPM2 activation and consequent disease exacerbation. While microglial TRPM2 has been definitively linked to worsened epilepsy (Table 4), the specific cell type and molecular pathways through which TRPM2 mediates other neurological disorders remain to be elucidated. Dysfunction of TRPV1 is observed in ASD, while TRPV1 expression is upregulated in neuropathic pain, epilepsy, anxiety, and depression (Table 5). This indicates that while TRPV1 is indispensable for physiological neuronal function, pathological stimuli drive its activation, thereby exacerbating disease progression. Moreover, TRPV1 shows a dual role in neurological disorders, including stroke, MS, AD, and PD (Table 5). This paradox may be explained by several factors: (1) Distinct pathological mechanisms in different rodent models, leading to model‐specific outcomes upon TRPV1 modulation; (2) The inherent bidirectional regulatory capacity of TRPV1 in neuroinflammatory processes, which can either exacerbate or resolve inflammation [194]; (3) The spatiotemporal specificity of TRPV1 expression and activity across different cell types and disease stages; (4) Divergent agonist or antagonist dosages and treatment durations across studies may lead to different therapeutic effects. Despite these complexities, TRPV1 is emerging as a novel therapeutic target for neurological diseases. TRPV4 activation generally promotes inflammation across a range of neurological conditions, including neuropathic pain, ischemic stroke, MS, PD, ASD, epilepsy, and depression (Table 5). Although expressed broadly in the nervous system, TRPV4 may exert its strongest pathogenic influence by reshaping microglial responses. Future study should clarify how microglial TRPV4 contributes to the pathology of neurological diseases.

TRPA1, TRPM2, TRPV1, and TRPV4 are involved in the neuroimmune processes of various neurological diseases, including neuropathic pain, migraine, AD, and epilepsy (Tables 2, 4, and 5), making them important therapeutic targets for these diseases. Capsaicin (for TRPV1) and miconazole (for TRPM2), two FDA‐approved drugs, show great potential in clinical studies. Besides these FDA‐approved drugs, the therapeutic potentials of TRP channels are implicated by various strategies, including antagonists, agonists, gene editing, and neuromodulation techniques. Antagonists such as HC‐030031 suppress TRPA1 to reduce immune response in nervous system and have therapeutic potentials for the intervention of neuropathic pain and AD [38, 43] (Table 2). ACA and 2‐APB reversed the symptoms of migraine and PD by inhibiting TRPM2 [59, 67] (Table 4). HC‐067047, an antagonist of TRPV4, exhibits therapeutic potentials by reducing microglia or astrocytes activation, and decreasing expressions of IL‐1β and IL‐6 in neuropathic pain, epilepsy, ASD, and depression [36, 138, 143, 146] (Table 5). New technologies such as nanotechnology‐based TRPV1‐targeting strategy enhances α‐synuclein clearance in microglia of PD models [119] (Table 5). Gene editing approaches for specific TRP channel member also shows notable effectiveness in different diseases (Tables 2, 3, 4, 5). Despite their promise, gene editing therapies for TRP channels face challenges due to ethics issues, unknown side effects, and long‐term risks, limiting clinical translation. Brain stimulation strategies (EA, rTMS, and TENS) have demonstrated promising therapeutic effects in neuropathic pain [91, 93] (Table 5). EA treatment shows beneficial effects for neuropathic pain, ischemic stroke, PD, and epilepsy by modulating TRPV family [31, 48, 91, 120, 139] (Table 5). However, the lack of specificity and highly varied effects in different individuals hinder the development of brain stimulations as TRP‐targeted therapies. Therefore, drug therapy is currently the main direction for the development of TRP‐targeted therapeutic strategies.

Despite the therapeutic promise, targeting TRP channels poses significant challenges. The TRP channel family comprises several isoforms with structural and functional similarity, which may cause unforeseen effects in the development of specific pharmacological agents. Thus, one of the major challenges in developing TRP channel drugs is to design pharmacological agents with high subtype specificity to minimize the off‐target effects. Although several high‐resolution structures of TRP channels have been obtained by cryo‐electron microscopy in recent years [195, 196, 197], the structural biology behind TRP channels is still a crucial factor in drug design and optimization. Addressing these challenges necessitates interdisciplinary collaborations, including synergistic work in systems neuroscience, cellular biology, and computational modeling, which together help to understand the TRP channel functions and regulatory mechanisms from different perspectives. While TRPA1, TRPM2, TRPV1, and TRPV4 are currently prioritized as therapeutic targets, their therapeutic exploitation requires novel strategies to ensure neuroprotection and minimal off‐target effects. Overcoming these obstacles will be pivotal for advancing TRP channel‐based anti‐neuroinflammation therapies for interventions in neurological disorders.

## Author Contributions

Conceptualization: Daji Guo, Lizhang Zeng, Lei Shi, and Shiqing Zhang. Funding acquisition: Lei Shi and Shiqing Zhang. Investigation: Daji Guo, Mengjiao Cai, and Yinyin Chen. Methodology: Daji Guo. Project administration: Lizhang Zeng, Lei Shi, and Shiqing Zhang. Supervision: Shiqing Zhang. Validation: Yanmin Xian and Chun Hu. Visualization: Daji Guo and Mengjiao Cai. Writing – original draft: Daji Guo, Mengjiao Cai, Yanmin Xian, Yinyin Chen, and Yujun Feng. Writing – review and editing: Daji Guo, Mengjiao Cai, Lizhang Zeng, Lei Shi, and Shiqing Zhang.

## Funding

This work was supported in part by the STI2030‐Major Projects (2022ZD0214400), the National Natural Science Foundation of China (82371175 and U24A20804), the International Science and Technology Cooperation Projects of Guangdong Province (2023A0505050121), Guangdong Basic and Applied Basic Research Foundation (2022B1515130007, 2023B1515040015, and 2023A1515030012), the Science and Technology Program of Guangzhou (202102070001).

## Conflicts of Interest

The authors declare no conflicts of interest.

## Data Availability

Data sharing not applicable to this article as no datasets were generated or analyzed during the current study.
